# Ischemic Stroke Induced Area Postrema Syndrome With Intractable Nausea, Vomiting, and Hiccups

**DOI:** 10.7759/cureus.8630

**Published:** 2020-06-15

**Authors:** Dylan T Cohen, Catherine Craven, Ilya Bragin

**Affiliations:** 1 Neurology, St. Luke's University Health Network, Bethlehem, USA; 2 Neurology, Lewis Katz School of Medicine at Temple University, Philadelphia, USA

**Keywords:** area postrema, area postrema syndrome, intractable nausea vomiting hiccups, ischemic stroke, chemoreceptor trigger zone, aps, neuromyelitis optica spectrum disorder, nmosd, cva vomit, stroke

## Abstract

The area postrema (AP) is a small, circumventricular organ located in the dorsal medulla and is characterized by an anastomosed capillary network with no blood-brain barrier. It contains the chemoreceptor trigger zone for vomiting, which is activated by noxious stimuli in the blood. Lesions to the AP produce a clinical syndrome referred to as area postrema syndrome (APS), which is characterized by intractable nausea, vomiting, and hiccups. APS manifests frequently as neuromyelitis optica spectrum disorders (NMOSD), where antibodies attack aquaporin-4 receptors, which are found in abundance in the AP. Its vascular supply is delivered by the anterior spinal artery or, at times, by small vessel branches of the vertebral artery itself.

Ischemic stroke is the fifth leading cause of death in the United States; however, APS due to ischemic stroke has rarely been described. We present a case of a 62-year-old male with ischemic stroke in the cerebellum and brainstem, which produced intractable APS due to extension within his AP. He was treated with metoclopramide 10 mg four times daily and ondansetron 8 mg every eight hours, which relieved his symptoms. Recognizing that the patient’s intractable nausea and vomiting was attributable to AP involvement was valuable in limiting further extraneous workup and focusing on our medical management. Ischemic stroke should be considered in the differential for APS. Given the size of the AP, thin-cut high-resolution diffusion-weighted MRI is warranted in patients with clinical APS. Recognizing that intractable nausea and vomiting may be attributable to stroke is valuable in mitigating extraneous and ineffective medical management. The patient case we describe in our report further outlines these findings.

## Introduction

Area postrema syndrome (APS) is characterized clinically by persistent episodes of intractable nausea, vomiting, and hiccups. These symptoms can be attributed to a lesion to the area postrema (AP) or “vomiting center” of the medulla [1]. Although a relatively rare condition, numerous cases are well described throughout the literature. The majority of cases involve lesions due to demyelination in patients who have underlying neuromyelitis optica spectrum disorder (NMSOD), for which these symptoms are virtually pathognomonic [1]. While APS is known to result from lesions in the AP in a general sense, a review of the literature involving PubMed, Google Scholar, and Embase return no results for any specific stroke-related cases. We present a seldom reported case of APS attributed to a stroke directly within the AP.

## Case presentation

A 62-year-old male with a medical history of hypertension, poorly controlled diabetes, and stroke with residual mild cognitive impairment and expressive aphasia presented to the hospital complaining of abdominal pain, vomiting, and ambulatory dysfunction for the last two days. He had a preexisting mid-metatarsal amputation of the left foot and was on aspirin 81 mg daily. He stated that he felt generally weaker than normal and was unable to ambulate in the ED. A noncontrast CT of the head was negative on presentation, and he was subsequently admitted for the management of dehydration secondary to suspected viral gastroenteritis. His nausea and vomiting improved shortly after admission. However, on the first day of hospitalization, the patient was being assessed by the medicine team as well as physical therapy and was found to be leaning backward and to the right when standing or attempting to ambulate. His family confirmed at this time that he had been doing this at home for a few days and had experienced several falls recently.

**Figure 1:**
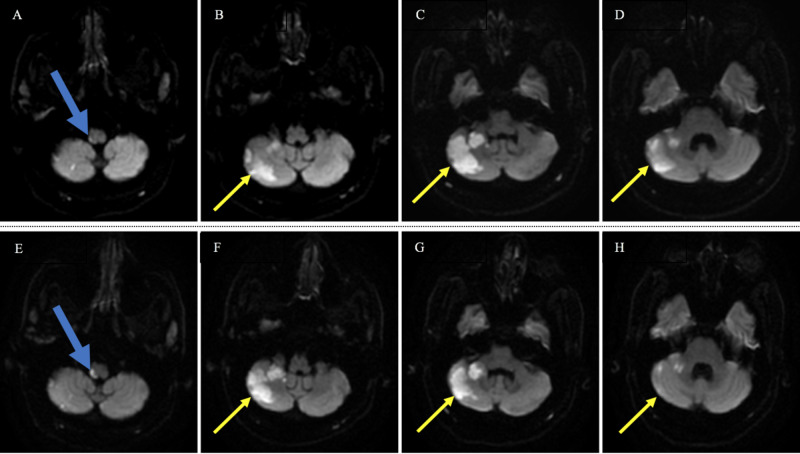
Initial and Secondary Stroke Images above and below each other correspond to the same anatomical plane. (A-D) Initial MRI: consecutive, rostral to caudal, axial DW MRI images with the blue arrow directed at hyperintensity representing small infarct in the midline-oriented area postrema; yellow arrows depict hyperintensity signifying the extent of full stroke extending from lateral medulla. (E-H) Repeat MRI: consecutive, rostral to caudal, axial DW MRI images with the blue arrow directed at hyperintensity representing worsening infarct in the region of area postrema; yellow arrows again represent regions of newly evolved stroke within the lateral medulla. DW, diffuse-weighted

Given these findings, the neurology service was consulted and he was assessed on his second day of hospitalization. He was noted to have left-sided dysmetria. A CT angiogram of the head and neck showed a congenitally hypoplastic left vertebral artery and otherwise patent vertebrobasilar system with multifocal intracranial stenosis in the anterior circulation bilaterally. MRI of the brain was performed and confirmed a large area of acute/subacute infarct in the right cerebellar hemisphere without mass effect (Figure 1A-1D). MRI at that time also showed restricted diffusion in a miniscule region of the AP on the right (Figure 1A). Echocardiogram performed showed an LVEF (left ventricular ejection fraction) of 60%, no regional wall motion abnormalities, normal wall thickness, and grade 1 diastolic dysfunction with trace aortic regurgitation. On day 5 of hospitalization, the patient became acutely more nauseous again and was noted to have projectile bilious vomiting. The nausea and vomiting lasted from minutes to hours and occurred with or without movement, typically following a meal. Notably, his neurologic examination was unchanged from the previous one, and the abdominal examination was unremarkable. The patient was sent for an abdominal X-ray obstruction series, which was negative. Subsequently, he was sent by the primary team for a repeat MRI of the brain to assess for worsening or a new infarct. The MRI was read as “stable” by the radiology service (Figure 1E-1H). He was initially treated with ondansetron 8 mg every six hours as needed, which was ineffective. The neurology service was reconsulted for input regarding the intractable nausea and vomiting. He also had concomitant hiccups. The MRI was reviewed by the neurology service, and it was apparent that the stroke in the AP had extended (Figure 1E) and explained his APS. The stroke etiology was felt to be likely secondary to vessel-to-vessel embolization due to chronic atherosclerotic disease and less likely. The patient was monitored on telemetry for several days without any events or abnormalities noted.

He was started on a scheduled antiemetic regimen of metoclopramide 10 mg four times daily and ondansetron 8 mg every eight hours. Within one day, the nausea and vomiting was under control, and the patient was able to eat again without difficulty and was successfully discharged to rehabilitation.

## Discussion

The AP is located within the dorsal vagal complex (DVC) of the medulla of the brainstem at the most caudal and posterior floor of the fourth ventricle [2]. Unlike several structures in the brain whose capillary systems are largely impermeable in conjunction with the blood-brain barrier, the AP does not fall under this classification. Rather, its position abutting the ventricular system labels it a circumventricular organ. Consequently, it is highly vascularized with several subregions of varied yet extensive capillary permeability, where blood flow is slowed, relative to the adjacent structures within the DVC, such as the dorsal motor nucleus of the vagus and nucleus tractus solitarius (NTS) [3]. This reduction in the rate of blood flow underlies its neuroendocrine importance in maximizing the time for chemical message exchange, allowing for optimal integration with the central nervous system [4]. Disruption of this system has implications in regulation of blood pressure, fluid balance, the immune system, feeding, and metabolism [4].

The role of the AP as a “vomiting center” has been well documented. The chemoreceptor trigger zone (CTZ) within the AP serves to detect emetogenic toxins in the capillary network and communicate with the NTS to eventually induce an emetic reflex [5]. When this area is damaged, episodic intractable nausea and vomiting occur. This physiological mechanism is seen in various clinical scenarios: chemotherapy and radiation, due to 5-HT3 receptor activation in the CTZ, as well as postoperative opiate use, in part due to D2 receptor activation. Ondansetron and phenothiazines, by their 5-HT3 and D2 antagonism, respectively, are effectively used as antiemetics in these presenting settings [5]. Emesis can be a common symptom in stroke, particularly in large strokes and those of the posterior fossa. Intractable hiccups, however, are significantly rarer, and their presence with intractable vomiting suggests a more localized lesion, especially involving the AP.

More commonly reported is APS due to inflammation, as in the case of NMSOD. The AP contains a high concentration of aquaporin-4 (AQP-4) water channel proteins, which are targeted in NMSOD. The AQP-4 antibody binding triggers complement activation and subsequently the downstream effects and histopathology seen in NMSOD. Patients also commonly present with blindness and paralysis due to AQP-4 protein density on optic nerves and spinal cord, whereas isolated APS attacks in AQP-4-positive NMOSD have a prevalence of 7.1-14.5% [6,7].

Although mechanisms of injury in AP lesions are not limited to inflammation, to our knowledge, vascular disruption in the form of stroke has not been chronicled. One case of isolated vomiting was initially reported due to infarction in the AP. Upon further review, however, the authors properly noted that the area of infarct was rather “a few millimeters above the area postrema” but believe there may have been an “undefined neural network” to explain the vomiting [8,9]. Nonetheless, other unique case reports evidence the recurring effects of AP injury as a mechanism for intractable nausea and vomiting. A 2010 report of a posterior inferior cerebellar artery compression on the AP details the case of a 12-year-old with chronic emesis who experienced relief of symptoms following a microvascular decompression [10]. A 2019 report details the case of a young woman with systemic lupus erythematosus (SLE) who endured an acute attack during a flare-up [11]. Like patients with NMSOD, SLE patients have been noted to have AQP-4 antibodies as well, although it is unknown if the patient in the case was tested for them. Our patient, albeit by rather unusual means, followed the trend of intractable nausea and vomiting due to comparable AP lesioning.

## Conclusions

Physician awareness of stroke extending into or isolated to the AP offers an opportunity for earlier intervention and directed action. AP strokes can be missed given the frequency of nausea and vomiting and should be considered appropriate settings, particularly if accompanied by intractable hiccups. A close review of imaging is critical as it is a small region and can be easily overlooked. This is important for both therapeutic purposes and limiting further potentially harmful and wasteful workup.
